# Salicylic Acid

**Published:** 1876-09

**Authors:** John A. Pritchett

**Affiliations:** Hayneville, Ala.


In this number of the Journal will be found a number of extracts from exchanges, giving the experience of members of the profession with salicylic acid in acute rheumatism. In almost every exchange received at this office for the past few months, we find something laudatory of the action of this new remedy in rheumatism. The following is the mode of administration suggested by Dr. Pritchett in the *American Medical Weekly:*

Hayneville, Ala., Aug. 16, 1876.

*Professor E. S. Gaillard:*

Dear Sir—I wish to give the readers of your *Weekly* the proper method of administering salicylic acid in acute rheumatism—namely, in the form of salicylate of ammonia. I find that the fluid drachm of the concentrated aqua ammonia neutralizes one drachm of the acid, developing considerable heat, and leaving a slight excess of ammonia. This excess is not at all objectionable, but rather advantageous, as combining the alkaline treatment with that by the salicylate. This makes a very concentrated solution, and a very palatable one, especially when diluted with some aromatic water and sweetened. Several cases occurring in the practice of myself and other physicians at this place, have received prompt and decided benefit from this agent. Respectfully,

JOHN A. PRITCHETT, M.D.

